# Molecular Mechanism of the Antiproliferative Activity of Short Immunostimulating dsRNA

**DOI:** 10.3389/fonc.2019.01454

**Published:** 2019-12-20

**Authors:** Mikhail I. Zharkov, Marina A. Zenkova, Valentin V. Vlassov, Elena L. Chernolovskaya

**Affiliations:** Laboratory of Nucleic Acids Biochemistry, Institute of Chemical Biology and Fundamental Medicine SB RAS, Novosibirsk, Russia

**Keywords:** immunostimulating dsRNA, antiproliferative activity, gene silencing, shRNA, pattern recognition receptors, PKR, RIG-I, IL-6

## Abstract

Small double-stranded RNAs with certain sequence motifs are able to interact with pattern-recognition receptors and activate the innate immune system. Recently, we identified a set of short double-stranded 19-bp RNA molecules with 3-nucleotide 3′-overhangs that exhibited pronounced antiproliferative activity against cancer cells *in vitro*, and antitumor and antimetastatic activities in mouse models *in vivo*. The main objectives of this study were to identify the pattern recognition receptors that mediate the antiproliferative action of immunostimulating RNA (isRNA). Two cell lines, epidermoid carcinoma KB-3-1 cells and lung cancer A549 cells, were used in the study. These lines respond to the action of isRNA by a decrease in the growth rate, and in the case of A549 cells, also by a secretion of IL-6. Two sets of cell lines with selectively silenced genes encoding potential sensors and signal transducers of isRNA action were obtained on the basis of KB-3-1 and A549 cells. It was found that the selective silencing of *PKR* and *RIG-I* genes blocked the antiproliferative effect of isRNA, both in KB-3-1 and A549 cells, whereas the expression of *MDA5* and *IRF3* was not required for the antiproliferative action of isRNA. It was shown that, along with *PKR* and *RIG-I* genes, the expression of *IRF3* also plays a role in isRNA mediated IL-6 synthesis in A549 cells. Thus, PKR and RIG-I sensors play a major role in the anti-proliferative signaling triggered by isRNA.

## Introduction

Oncological diseases are among the greatest challenges of modern medicine. Current approaches to cancer treatment, including surgical removal of tumor mass followed by chemotherapy or radiotherapy, are not always sufficiently effective due to incomplete removal of cancer cells resulting in disease recurrence and a high incidence of side effects. Thus, the development of new agents for antitumor therapy, combining low toxicity, and high efficiency, is required.

Carcinogenesis is often accompanied by genetic and epigenetic alterations, resulting in the expression of tumor antigens. Normally, the immune system can recognize these tumor antigens as foreign, but tumors escape from immunosurveillance by exploiting various mechanisms and suppressing the immune response. Immunotherapy is a highly potent approach for the therapy of neoplastic diseases, which allow the activation of the host immune system and elimination of tumor cells.

Long and short dsRNAs, depending on their sequence and structure, can activate the innate immune system through pattern recognition receptors (PRRs). These PRRs include Toll like receptors (TLRs), such as TLR3/7/8 (1–4), retinoic acid inducible gene-I (RIG-I), RIG-I-like helicase MDA5 (5, 6), dsRNA dependent protein kinase R (PKR) (7, 8), and NOD-like receptors such as NLRP3 and NOD2 (9, 10). After activation, these receptors, acting through signal transduction pathways, can induce apoptosis, proliferation blockage and induction of secretion of type I interferons and/or inflammatory cytokines by immune cells. Thus, these agents can have both a direct effect on the tumor and an indirect effect by activating the immune system.

The spectrum of biological effects induced by an immunostimulating agent in certain cells may differ depending on the receptors recognizing the agent and the activity of the signaling pathways determined by the pattern of gene expression of the cell (11, 12). Preferential secretion of type I interferons will have a pronounced antitumor effect; however, if immunostimulation is accompanied by a significant enhancement of pro-inflammatory cytokine synthesis, severe side effects will occur preventing the use of such agents for therapy. In particular, upregulation of TNFα synthesis leads to fever, apoptotic death of normal cells, cachexia, and inflammation. A high level of IL-6, another pro-inflammatory cytokine, causes inflammatory, and auto-immune processes in normal tissues (13).

Currently, immunostimulatory nucleic acids, such as long dsRNAs (14, 15), CpG-containing oligodeoxyribonucleotides (16–19), siRNAs with immunostimulatory motifs (20–24), oligoribonucleotides with 5′-terminal triphosphates (5, 25, 26), and chimeric molecules containing an immunostimulatory domain (27) are being investigated as potential adjuvants in antitumor immunotherapy. Previously, we identified a short 19-bp dsRNA with 3-nt 3′-overhangs, which possesses immunostimulatory properties (here and after the Immunostimulatory RNA is expressed as isRNA). The isRNA did not have any substantial homology with any human or mouse mRNA and, thus, does not change gene expression via an RNA interference mechanism. We have shown that isRNA displays pronounced antiproliferative activity with respect to tumor cells, induces interferon-α synthesis by PBMC (28, 29) and exhibits antitumor and antimetastatic effects *in vivo* in animal models of tumor progression (30, 31). isRNA is low in toxicity *in vivo*, because it does not stimulate a notable production of pro-inflammatory cytokines, including TNF-α (29).

The main objective of this study was to identify the PRRs that are responsible for isRNA recognition and that mediate its antiproliferative action with respect to KB-3-1 and A549 cells and the induction of IL-6 synthesis in A549 cells. We evaluated the effects of selective silencing of genes that encode PRRs on the antiproliferative activity of isRNA in KB-3-1 and A549 cell lines. The data obtained reveal that isRNA implements antiproliferative activity and induction of IL-6 synthesis by acting through PKR and RIG-I sensors.

## Materials and Methods

### Synthesis of isRNA

Oligoribonucleotides (strand 1: 5′-GUGUCAGGCUUUCAGAUUUUUU-3′; strand 2: 5′-AAAUCUGAAAGCCUGACACUUA-3′) were synthesized on an automated DNA/RNA synthesizer ASM-800 (Biosset, Russia) using ribo-β-cyanoethyl phosphite amides (GlenResearch, United States) and the published protocols (32) optimized for this device. After complete deblocking by standard methods, target products were isolated by electrophoresis in 12% PAAM/ 8M urea gel (acrylamide:N,N′-methylenebisacrylamide 30:0.5), using 0.1 M TBE as the running buffer at 30 V/cm. Bands corresponding to the target products were detected in the gel by UV shadow and excised; the oligonucleotides were eluted with 0.3 M NaAc (pH 5.2) and precipitated with ethanol as sodium salts. The purified oligoribonucleotides were characterized by MALDI-TOF-MS. isRNA was prepared by annealing strands 1 and 2 at 100 μM each in a buffer containing 15 mM HEPES/KOH (pH 7.4), 50 mM potassium acetate, 1 mM magnesium acetate, and subsequently stored at −20°C.

### Cell Cultures

Human epidermoid carcinoma KB-3-1 cells, lung carcinoma A549 cells and embryonic kidney HEK293T cells were purchased from the bank of cell cultures of the Institute of Cytology, Russian Academy of Sciences (St. Petersburg, Russia). A549 and KB-3-1 cells do not produce type I IFN, A549 is able to produce IL-6 after stimulation. The cells were cultured in DMEM medium supplemented with 10% fetal bovine serum (FBS), 100 U/ml penicillin, 0.1 mg/ml streptomycin, and 0.25 μg/ml amphotericin at 37°C in a humidified atmosphere with 5% CO_2_ (standard conditions).

### RNA Isolation and RT-qPCR

Total RNA was isolated from the cells using the standard Trizol method (33). The reverse transcription (RT) reaction was carried out using the RT2 First Strand Kit (QIAGEN, Germany) according to the manufacturer's protocol. The resulting cDNA was amplified in a reaction mixture of 20 μl, containing 1 μl of cDNA, 0.25 μM specific primers (Table 1) and Biomaster HS-qPCR SYBR Blue (2×) master mix (BioLab Mix, Russia) containing SYBR-Green fluorescent dye and hot start DNA polymerase. Real-time PCR was carried out using an iQ5 amplifier (Bio-Rad, USA) according to the following scheme: one cycle−3 min, 95°C; 40 cycles−30 s, 95°C, 30 s−58°C; and 30 s−72°C. The mRNA level of the specific genes was normalized to the level of *GAPDH* mRNA. All measurements were done in triplicates.

**Table 1:** Sequences of specific primers used in qPCR.

**Specific primer**	**Sequence**
GAPDH forward	5′-CCCCAATGTGTCCGTCGTG-3′
GAPDH reverse	5′-GCCTGCTTCACCACCTTCT-3′
TLR3 forward	5′-GTCTCACCTCCACATCCTTA-3′
TLR3 reverse	5′-CCCGAAAACCTTCTTCTCAA-3′
TLR7 forward	5′-TCTTCAACCAGACCTCTACATTCCA-3′
TLR7 reverse	5′-GGAACATCCAGAGTGACATCACAG-3′
TLR8 forward	5′-GCTGCTGCAAGTTACGGAATGA-3′
TLR8 reverse	5′-GATTGCTGCACTCTGCAATAACTGA-3′
IRF3 forward	5′-CTTTTCCCAGCCAGACAC-3′
IRF3 reverse	5′-CCAACACCATGTTACCCAG-3′
IRF7 forward	5′-CAGAGCCGTACCTGTCAC-3′
IRF7 reverse	5′-GAATGCTACCTGCTGGGG-3′
RIG-I forward	5′-GGACAAAAGGGGAAAGTTGT-3′
RIG-I reverse	5′-GTTCACAAGAATCTGTGGAGT-3′
NOD2 forward	5′-GTTCAACCTCAAGGGCTTC-3′
NOD2 reverse	5′-ATCTGTAGTGGTCTTTGGGG-3′
MDA5 forward	5′-ACATAACAGCAACATGGGCAGTG-3′
MDA5 reverse	5′-TTTGGTAAGGCCTGAGCTGGAG-3′
PKR forward	5′-TGCGATACATGAGCCCAGAACAGA-3′
PKR reverse	5′-ATGCCATCCCGTAGGTCTGTGAAA-3′

### Generation of shRNA-Expressing Lentiviral Vectors and Transduction of Target Cells

The lentiviral vectors were constructed by cloning synthetic insertions, encoding specific shRNA, into the pGPV vector (Evrogen, Russia) under the H1 promoter, between 4,605 bp and 4,614 bp (BamHI and EcoRI restriction sites, respectively) according to standard genetic engineering techniques. The lentiviral vector encoded the reporter fluorescence protein copGFP to visualize the transduced cells. The sequences of insertions, encoding shRNA, were selected using an RNAi Design Tool program (34) (Table 2).

**Table 2:** Sequences of insertions encoding shRNA, targeting *IRF3/7, TLR7/8, NOD2, RIG-I, MDA5*, and *PKR* genes, and shRNA with scrambled sequence.

**Designation**	**Sequence of shRNA encoding insertion**
Scr	5′-p-GATCCCAAGTCTCGTATGTAGTGGTTCAAGAGACCACTACATACGAGACTTGTTTTTG-3′ 3′-GGTTCAGAGCATACATCACCAAGTTCTCTGGTGATGTATGCTCTGAACAAAAACTTAA-p-5′
TLR7 (4,579–4,597)	5′-p-GATCCGGAGAAGAAACCAAAGTTTTTCAAGAGAAAACTTTGGTTTCTTCTCCTTTTTG-3′ 3′-GCCTCTTCTTTGGTTTCAAAAAGTTCTCTTTTGAAACCAAAGAAGAGGAAAAACTTAA-p-5′
TLR8 (579–597)	5′-p-GATCCGGGCATTTCAAGACTTATATTCAAGAGATATAAGTCTTGAAATGCCCTTTTTG-3′ 3′-GCCCGTAAAGTTCTGAATATAAGTTCTCTATATTCAGAACTTTACGGGAAAAACTTAA-p-5′
PKR (2,133–2,152)	5′-p-GATCCCGACCTAACACATCTGAAATTCAAGAGATTTCAGATGTGTTAGGTCGTTTTTG-3′ 3′-GGCTGGATTGTGTAGACTTTAAGTTCTCTAAAGTCTACACAATCCAGCAAAAACTTAA-p-5′
MDA5 (3,253–3,272)	5′-p-GATCCGTGCCGACTATCAAATAAATTCAAGAGATTTATTTGATAGTCGGCACTTTTTG-3′ 3′-GCACGGCTGATAGTTTATTTAAGTTCTCTAAATAAACTATCAGCCGTGAAAAACTTAA-p-5′
RIG I (1,103–1,122)	5′-p-GATCCAGACATGGGTATAGAGTTATTCAAGAGATAACTCTATACCCATGTCTTTTTTG-3′ 3′-GTCTGTACCCATATCTCAATAAGTTCTCTATTGAGATATGGGTACAGAAAAAACTTAA-p-5′
NOD2 (926–944)	5′-p-GATCCCAAATGCCACCAGGAACTGTTCAAGAGACAGTTCCTGGTGGCATTTGTTTTTG-3′ 3′-GGTTTACGGTGGTCCTTGACAAGTTCTCTGTCAAGGACCACCGTAAACAAAAACTTAA-p-5′
IRF3 (600–618)	5′-p-CCGGGATCTGATTACCTTCACGGAACTCGAGTTCCGTGAAGGTAATCAGATCTTTTTG-3′ 3′-CTAGACTAATGGAAGTGCCTTGAGCTCAAGGCACTTCCATTAGTCTAGAAAAACTTAA-p-5′
IRF7 (1,298–1,317)	5′-p-GATCCGCACGTTCCTATACGGCCCTTCAAGAGAGGGCCGTATAGGAACGTGCTTTTTG-3′ 3′-GCGTGCAAGGATATGCCGGGAAGTTCTCTCCCGGCATATCCTTGCACGAAAAACTTAA-p-5′

The transient co-transfection of HEK293 T cells with shRNA-expressing lentiviral plasmid and packaging plasmids (Invitrogen, USA) was carried out using Lipofectamin 2000 (Invitrogen) according to the manufacturer's protocol. Viral stocks were collected 3 days after transfection. Obtained viruses were used for the transduction of KB-3-1 and A549 cells using polybrene at a final concentration of 10 μg/ml. Three days after transduction the cells were subjected to FACS analysis and sorting.

### FACS

Transduced cells were isolated using a FACSAria (BD, USA) in the single cell mode at the appropriate sort rate (e.g., below 100 cells per second). Cell sorting was performed using fluorescent protein copGFP expressed in the target cells as a reporter. Parental cell lines were used to determine the background fluorescence in such a way that <1% of non-fluorescent cells were included in the sort gate.

### Cell Proliferation Assay

The relative number of living cells (normalized cell index) was monitored in real time using the xCELLigence RTCA DP Analyzer (ACEA Bioscience, USA). One day before transfection, the cells were plated in a 16-well E-Plate (ACEA Bioscience, USA) (at densities of 1 × 10^3^ cells per well for KB-3-1 or 1.5 × 10^3^ cells per well for A549 in 100 μl), and allowed to adhere overnight under standard conditions. On the day of the experiment the cells were transfected with isRNA using cationic liposomes 2X3-DOPE (35). Complexes of cationic liposomes with isRNA were preformed in a serum-free Opti-MEM medium by mixing equal volumes of solutions of 2X3-DOPE and isRNA, followed by incubation for 20 min at room temperature.

isRNA/2X3-DOPE complexes used for KB-3-1 cells were prepared using N/P = 1/4, with a final concentration in the well of 2.2 μM for 2X3-DOPE and 50 nM for isRNA. isRNA/2X3-DOPE complexes using A549 cells were prepared using N/P = 1/6 with a final concentration in the well of 6.6 μM for 2X3-DOPE and 100 nM for isRNA. The resulting complexes, in a volume of 50 μL, were added to the cells growing in 100 μl of medium. Cells were incubated with the complexes under standard conditions for 4 h. Then, the culture medium was replaced by fresh medium. The cells were allowed to grow under standard conditions for 96 h. Data were recorded every 4 h for 96 h post transfection, and were expressed as mean values of measurements in four wells ± SD.

### IL-6 Level Measurement

An ELISA was used to measure the IL-6 level in culture medium containing A549 cells stimulated with isRNA. One day before transfection, A549 cells were plated in a 24-well plate (at densities of 5 × 10^4^ cells per well) and allowed to adhere overnight under standard conditions. On the day of the experiment, cells were transfected with isRNA as described above. The cells were incubated under standard conditions for 20 h. The IL-6 level in culture medium was measured by ELISA using Interleukin-6-EIA-BEST (Vector-Best, Russia), according to the manufacturer's protocol, and the data were expressed as mean values of measurements in two wells ± SD.

### Cell Cycle Analysis

For the analysis of cell cycle progression, KB-3-1 cells were plated in a 24-well plate (at densities of 2 × 10^4^ cells per well) and allowed to adhere overnight under standard conditions. On the day of the experiment, cells were transfected with isRNA as described above. The cells were incubated under standard conditions for 48 h. Then cells were stained using Hoechst 33342 Ready Flow™ Reagent (R37165, Thermo Fisher Scientific, USA), according to the manufacturer's protocol. Stained cells were analyzed utilizing flow cytometer NovoCyte 3000 (ACEA Bioscience, USA) and NovoExpress Software. A total of 10,000 cells were analyzed from each sample. The described above apoptosis and cell cycle analyses were carried out two times in independent experiments, and the data were expressed as mean values ± SD.

### Statistical Analysis

The statistical significance of the differences in experiments was determined using the two-tailed Student's *t*-test (data are expressed as means ± SD). Differences were considered statistically significant for *p* < 0.05.

## Results

### Selection of Potential Mediators of isRNA Antiproliferative Action and Cell Models

Two cell lines, epidermoid carcinoma KB-3-1 cells and lung cancer A549 cells were used to identify sensors mediating isRNA antiproliferative activity in this study, because, as highlighted above, it has been shown that isRNA inhibits proliferation of these cells (28, 29). Moreover, A549 cells also secreted IL-6 in response to isRNA and, therefore, A549 cells can be used to evaluate both the direct antiproliferative effect and the antiproliferative effects mediated by cytokine secretion.

As a first step, we selected cytoplasmic receptors RIG-I, MDA5, NOD2, and PKR, and the interferon regulatory transcription factor IRF3/7 as potential mediators or sensors of isRNA antiproliferative activity based on data in the literature (5, 7–10, 36, 37) and estimated the expression levels of the genes encoding potential mediators of isRNA action in the KB-3-1 and A549 cell lines to assess the possibility of their participation in the signal transduction in these lines.

Relative levels of mRNA encoded potential isRNA sensors and signal transducers were measured in KB-3-1 and A549 cells by qRT-PCR with specific primers (Table 3). It can be seen that KB-3-1 cells had a high level of *PKR* mRNA and average levels of *IRF3, MDA5, RIG-I* mRNA. Expression of *TLR3/7/8, NOD2*, and *IRF7* was not detected in these cells. A549 cells also had a high level of *PKR*, average levels of *IRF3, MDA5, RIG-I*, and a very low level of *TLR3* mRNA. Levels of *TLR7/8, NOD2* and *IRF7* mRNA were below the detection limit. It should be noted that the relative levels of the studied mRNA in KB-3-1 normalized to *GAPDH* mRNA were 2–6 fold higher than those in A549 cells.

**Table 3:** Relative mRNA level of potential isRNA sensors and signal transducers in KB-3-1 and A549 cells.

**Potential sensor of isRNA**	**Normalized mRNA level**^**a**^ **× 10**^****3****^	
	**KB-3-1**	**A549**
IRF3	8.5 ± 0.5	5.0 ± 0.9
IRF7	n. d.^b^	n. d.
MDA5	2.8 ± 0.4	0.8 ± 0.2
NOD2	n. d.	n. d.
PKR	90.6 ± 5.6	13.6 ± 2.1
RIG-I	6.1 ± 0.3	2.1 ± 0.3
TLR3	n. d.	0.5 ± 0.1
TLR7	n. d.	n. d.
TLR8	n. d.	n. d.

a *The mRNA level was measured by qRT-PCR and normalized to the GAPDH mRNA level. Experiments were performed in triplicate. The data represent means ± SD*.

b *n.d.—relative mRNA level is below the reliable detection limit*.

Thus, based on mRNA levels, PKR, IRF3, MDA5, and RIG-I proteins can be present in the cells and participate in the recognition of isRNA or/and isRNA signal transduction in both cell lines. Therefore, these mRNAs were chosen as targets for RNAi to elucidate the role of the corresponding proteins in the antiproliferative process.

### Silencing of Genes Encoding Potential Mediators of isRNA Antiproliferative Action by shRNA

The proteins considered as participants in the signaling cascades have different stability and half-lifetimes, therefore, in order to reliably reduce their level in the studied cells during the experiment, it is necessary to achieve a sustained reduction in the level of the corresponding mRNA. For this purpose, we used short hairpin RNA (shRNA) instead of traditional siRNA and obtained cell sublines stably expressing particular shRNA. Recombinant lentiviruses were used as a vector for delivery of shRNA-encoding sequences (Table 2) to the cell genome. A vector containing shRNA with a scrambled sequence was used as a control of specificity.

A549 and KB-3-1 cells were transduced by shRNA-expressing lentiviruses with the transduction level, according to the expression of reporter fluorescent protein copGFP, of at least 70–90%. Then, cell sorting was performed in such a way that <1% of non-fluorescent cells were present in the final population. The relative mRNA level of silenced genes in the obtained cell sublines was measured by qRT-PCR. It is seen (Table 4) that, in the sublines, expression of the silenced genes is significantly reduced (from 55 to 94% in comparison with parent cells). Maximum inhibition levels were observed for *MDA5* and *RIG-I* in A549 (93 and 94% respectively). Moreover, the inhibition of the studied genes in A549 cells was higher than those in KB-3-1 cells, which may be explained by the fact that the initial expression levels of the corresponding mRNAs were lower in these cells. It should be noted that suppression of gene expression was observed only under specific shRNA, expression of other target genes in the individual cell lines expressing shRNA, directed to one of the target genes, did not change. PKR, RIG-I, MDA5 silencing in A549 sublines at the protein level was shown by us previously by western blot analysis (38). Thus, we obtained A549 and KB-3-1 cell sublines with selectively silenced *IRF3, MDA5, PKR*, and *RIG-I* genes to study the participation of proteins encoded by inhibited genes in signaling pathways activated by isRNA.

**Table 4:** Inhibition of the expression of PRRs and transcription factors by shRNA in transduced KB-3-1 and A549 cell lines.

**Cell line**	**Target mRNA**	**Relative mRNA level, % of control^**a**^**
KB-3-1-MDA5	*MDA5*	45 ± 2
KB-3-1-IRF3	*IRF3*	32 ± 2
KB-3-1-RIG-I	*RIG-I*	36 ± 3
KB-3-1-PKR	*PKR*	14 ± 3
A549-MDA5	*MDA5*	7 ± 2
A549-IRF3	*IRF3*	22 ± 2
A549-RIG-I	*RIG-I*	6 ± 2
A549-PKR	*PKR*	18 ± 7

a *The relative mRNA level was measured by qRT-PCR. The relative level of mRNA of target genes in parent and scrambled-shRNA-expressing KB-3-1 and A549 cell sublines was set at 100%. GAPDH mRNA was used as internal standard. Experiments were performed in triplicate. The data represent mean ± SD*.

### The Effect of Specific Gene Silencing on the Antiproliferative Activity of isRNA in KB-3-1 and A549 Cell Sublines Stably Expressing shRNAs

**Figure 1:**
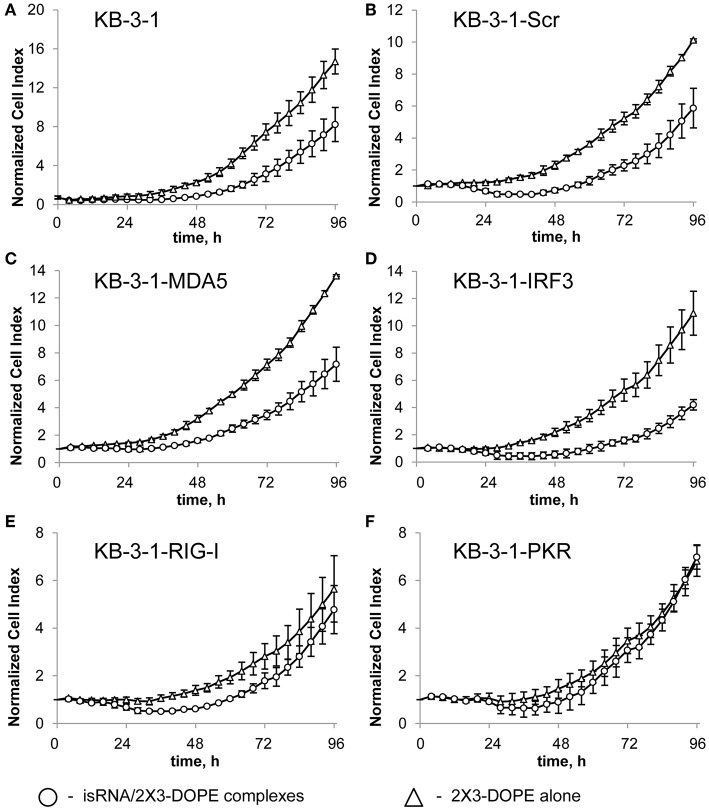
The effect of isRNA/2X3-DOPE complexes (◦) or 2X3-DOPE alone (Δ) on the proliferation of parent KB-3-1 cells **(A)**, and sublines KB-3-1-Scr **(B)**, KB-3-1-MDA5 **(C)**, KB-3-1-IRF3 **(D)**, KB-3-1-RIG-I **(E)**, and KB-3-1-PKR **(F)**. After transfection, the relative number of living cells was measured every 4 h for 96 h. The number of living cells 4 h after transfection was set at 1. Experiments were performed in four repeats. The data represent means ± SD.

To identify the sensors mediating the antiproliferative activity of isRNA on the cancer cells, we investigated the effect of PRR gene silencing on the antiproliferative activity of isRNA using the obtained KB-3-1 and A549 sublines. The relative number of living cells (normalized cell index) in real time was monitored every 4 h using the xCELLigence RTCA DP Analyzer, starting 4 h after transfection with isRNA (Figure 1). The data (Figure 1; Supplementary Figure S1) are displayed as time dependence of Normalized Cell Index (the relative number of living cells, normalized to the number of living cells 4 h after transfection in the same cell sublines) of cells, treated with isRNA/2X3-DOPE lipoplexes and 2X3-DOPE only. It is seen (Figure 1) that isRNA significantly inhibits proliferation of parental KB-3-1 cells. The decrease in the number of living cells under isRNA treatment of the KB-3-1-Scr was comparable with those in the parental cell line. The cells with selectively inhibited *MDA5* and *IRF3* (KB-3-1-MDA5, KB-3-1-IRF3) were also as sensitive to the antiproliferative action of isRNA as the parent cell line. On the contrary, KB-3-1-RIG-I and KB-3-1-PKR cells with downregulated *PKR* and *RIG-I*, respectively, did not respond to isRNA, and the growth rate of these cells did not differ reliably from the proliferation rate of the cells treated with 2X3-DOPE only.

An analysis of the action of isRNA on A549 cell sublines (Supplementary Figure S1; Table 5) showed similar dependences. In particular, cell treatment with isRNA resulted in a decrease in cell proliferation in parental A549 cells, A549-Scr, A549-MDA5, and A549-IRF3 cells, but did not reliably reduce the proliferation rate of A549-RIG-I and A549-PKR cells. A quantitative comparison of the antiproliferative effect of isRNA—the decrease in the number of living cells 96 h after transfection with isRNA/2X3-DOPE compared to mock-treated cells—was performed. The relative number of cells treated with 2X3-DOPE alone was set at 100%. It was shown (Table 5, Figure 1, and Supplementary Figure S1) that isRNA effectively inhibited proliferation in parental KB-3-1 and A549 cells to 44 ± 11% and 47 ± 7%, respectively. The antiproliferative effects of isRNA on the cells expressing Scr-shRNA were comparable with the effects of isRNA on parental cell lines (41 ± 11% and 62 ± 5% for KB-3-1-Scr and A549-Scr, respectively). The cells with selectively inhibited *MDA5* and *IRF3* (KB-3-1-MDA5, KB-3-1-IRF3, A549-MDA5, and A549-IRF3), were as sensitive to the antiproliferative effects of isRNA as parent cell lines (47 ± 7, 61 ± 5, 68 ± 11, and 63 ± 11%, respectively). On the contrary, silencing of *PKR* and *RIG-I* genes significantly reduces the antiproliferative effect of isRNA. The growth rate of KB-3-1-RIG-I, KB-3-1-PKR, A549-RIG-I, and A549-PKR cells after isRNA treatment did not differ reliably from the proliferation rate of the cells treated with 2X3:DOPE only. It is worth mentioning that both in KB-3-1 and A549 cell sublines, the effects of isRNA were similar (Table 5).

**Table 5:** The effect of PRRs gene silencing by shRNA on the antiproliferative activity of isRNA in KB-3-1 and A549 cell lines and sublines.

**Cell line**	**Silenced gene**	**Antiprolefirative effect^**a**^, %**
KB-3-1	^*^	44 ± 11
KB-3-1-Scr	^*^	41 ± 11
KB-3-1-MDA5	*MDA5*	47 ± 7
KB-3-1-IRF3	*IRF3*	61 ± 5
KB-3-1-RIG-I	*RIG-I*	8 ± 8
KB-3-1-PKR	*PKR*	2 ± 4
A549	^*^	35 ± 7
A549-Scr	^*^	50 ± 8
A549-MDA5	*MDA5*	68 ± 11
A549-IRF3	*IRF3*	63 ± 11
A549-RIG-I	*RIG-I*	9 ± 7
A549-PKR	*PKR*	8 ± 8

* *no genes were silenced*.

a *Antiproliferative effect (%)—the decrease in the number of living cells 96 h after transfection with isRNA/2X3-DOPE compared to the mock-treated cells. The relative number of cells treated with 2X3-DOPE alone was set at 100%*.

It should be noted (Tables 4, 5) that the inhibition level of *RIG-I* in KB-3-1-RIG-I was lower than the inhibition level of *PKR* in KB-3-1-PKR (64 and 86%, respectively), and the inhibition level of *RIG-I* in A549-RIG-I was higher than the inhibition level of *PKR* in A549-PKR (94 and 82%, respectively). However, for all of these sublines comparable levels of antiproliferative effects mediated by isRNA were observed: 8, 2, 9, and 8% for KB-3-1-RIG-I, KB-3-1-PKR, A549-RIG-I, and A549-PKR, correspondingly showing no sensitivity toward isRNA.

We assume that proteins whose genes were silenced in the cellular sublines, which retained sensitivity to the anti-proliferative action of isRNA, do not interact with or do not participate in the signal transduction from isRNA, leading to a significant decrease in their proliferation rate. On the contrary, the inhibited proteins in the sublines which have lost sensitivity to isRNA are mediators of isRNA antiproliferative activity. Thus, the obtained data show that isRNA implements its antiproliferative activity though PKR and RIG-I sensors.

### The Effect of Gene Silencing on the Cytokine-Inducing Activity of isRNA in A549 Cell Sublines

**Figure 2:**
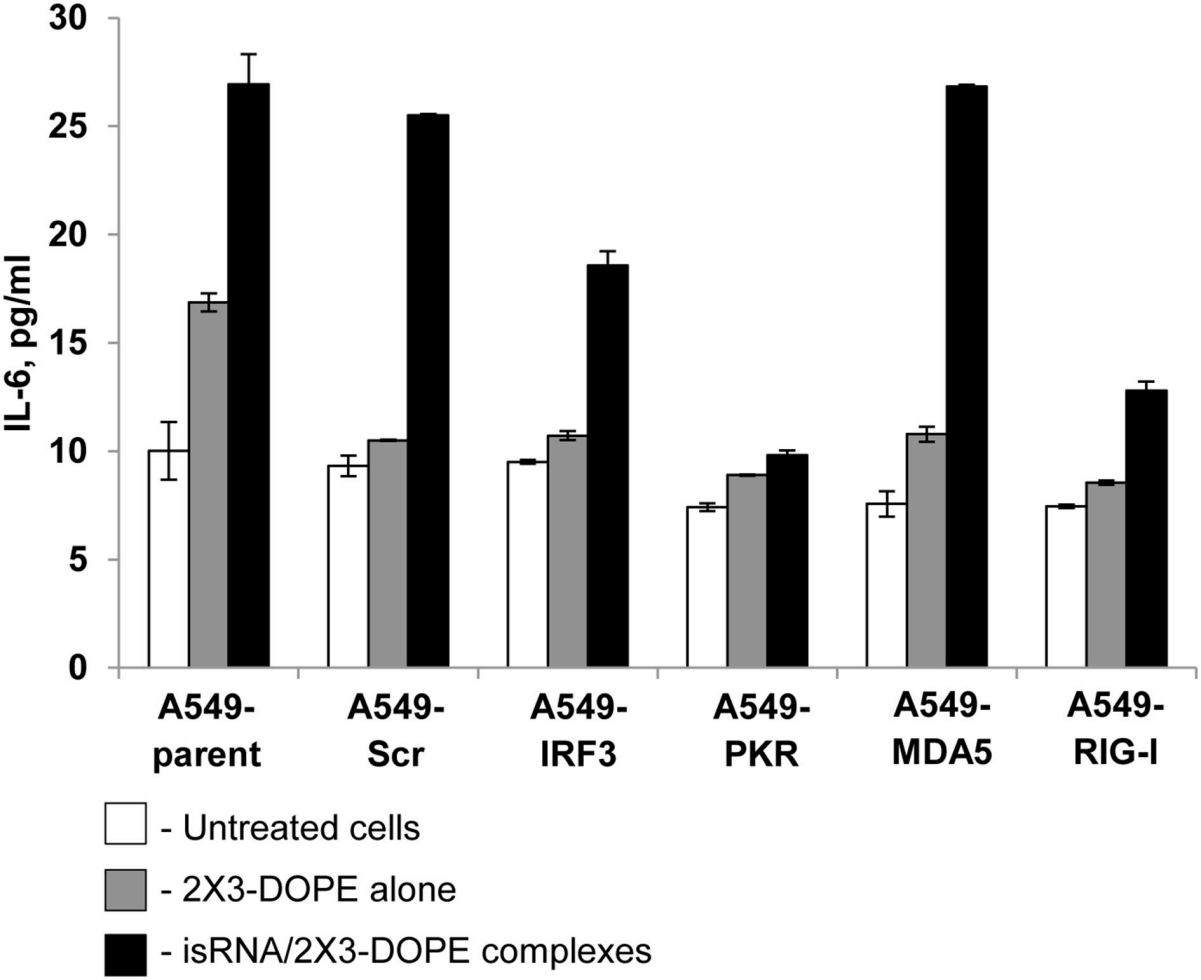
IL-6 levels in A549 cell sublines after transfection with isRNA/2X3-DOPE complexes (black bars) or 2X3-DOPE alone (gray bars) compared to untreated cells (white bars). The IL-6 level was determined by ELISA 24 h after transfection. Experiments were performed in duplicate. The data represent means ± SD.

We investigated the effect of silencing particular genes on the production of IL-6 in response to isRNA in A549 sublines, to determine the PRR responsible for the cytokine-inducing activity of isRNA. The cells were transfected with isRNA/2X3-DOPE lipoplexes (35), and levels of IL-6, produced by different A549 sublines in response to isRNA, were measured by Interleukin-6-EIA-BEST 24 h after transfection. Parent A549 cells produced IL-6 in response to isRNA treatment (Figure 2); the IL-6 level in the medium of treated cells was 3 times higher than the IL-6 level produced by untreated cells (27 ± 1.4 pg/ml and 10 ± 1.3 pg/ml for treated and untreated cells, respectively). The activation of IL-6 production by isRNA in A549-Scr and A549-MDA5 cells was similar to the IL-6 level in isRNA-treated parental cells (26 ± 0.06 pg/ml and 27 ± 0.09 pg/ml, respectively). Silencing of *IRF3* resulted in a less pronounced activation by isRNA of IL-6 synthesis in A549-IRF3 cells (19 ± 0.67 pg/ml) in comparison with controls (A549, A549-Scr) and A549-MDA5 cells.

Silencing of *RIG-I* and *PKR* by shRNA blocked the activation of IL-6 synthesis by isRNA. IL-6 levels in A549-RIG-I and A549-PKR cells were 13 ± 0.41 and 10 ± 0.23 pg/ml, respectively, which was similar to the IL6 level in the untreated or mock-treated cells (Figure 2). The data obtained show that RIG-I and PKR receptors play a major role in the induction of IL-6 synthesis in A549 cells in response to isRNA. Also, IRF3 could participate in signal transduction from isRNA, triggering activation of IL6 synthesis.

### The Effect of Gene Silencing on the isRNA Induced Retardation of Cell Cycle Progression

**Figure 3:**
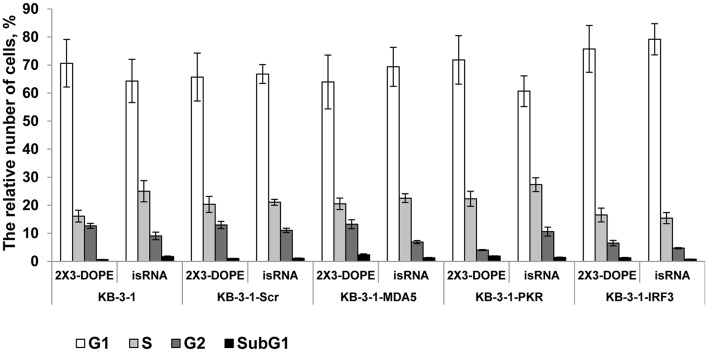
The effect of isRNA/2X3-DOPE complexes (isRNA) or 2X3-DOPE alone (2X3-DOPE) on retardation of cell cycle progression of KB-3-1 cell sublines. The cell cycle distribution was analyzed 48 h after transfection. The data expressed as a relative number of cells from total population related to different cell cycle stages. Experiments were performed in two repeats. The data represent means ± SD.

We previously showed that isRNAs (100 nM) delivered to the cells by Lipofectamine 2000 induce cell growth arrest in KB-3-1 cell line rather than cell death by apoptosis (29). We investigated the effect of particular genes silencing on the isRNA induced retardation of cell cycle progression in KB-3-1 sublines, to determine the PRR responsible for the antiproliferative activity of isRNA. The cells were transfected with isRNA/2X3-DOPE lipoplexes (35), and the distribution of cells between cell cycle stages were measured by flow cytometry after staining with Hoechst 33342 Ready Flow™ Reagent, 48 h after transfection. isRNA concentration 50 nM was used, with no observed toxicity or cell death. The use of a lower concentration for studying the antiproliferative effect was required due to the application of a more efficient transfection agent (2 × 3-DOPE) in the present study. All cells grown in the experimental wells were collected, including floating cells. It was found (Figure 3) that the number of cells in the subG1 population in all sublines was insignificant, which does not indicate the induction of apoptosis when using isRNA at a concentration of 50 nM. The obtained data show, that the number of parental KB-3-1 cells in G1-phase does not alter significantly in the presence of isRNA/2X3-DOPE lipoplexes, while the number of cells in S-phase increases, simultaneously the number of cells in G2-phase decreases, which indicates the blocking of cell division in cells under the action of isRNA. Similar changes in the distribution of cells over the phases of the cell cycle were also observed for subline cells with the *MDA5* and *IFR3* genes silencing, in which there was a decrease in the number of cells in the G2 phase under isRNA action. Another situation was observed for a cell line with inhibited PKR: the number of cells in the G2 phase in the cells treated only with a transfection agent was lower than in other sublines, however, under the action of isRNA it did not decrease, but even increased, which confirms what we obtained using the antiproliferation test evidence that blocking *PKR* gene expression prevents the antiproliferative effect of isRNA in this cell line. The data obtained confirms that PKR sensor play a major role in the antiproliferative effect of isRNA.

## Discussion

Several groups of PRR can recognize exogenous nucleic acids. The first group of receptors includes TLRs 3/7/8/9 (36), which are localized in the endosomal membranes or on the cell surface. It was shown that TLR9 recognizes unmethylated CpG DNA, while TLR7 and TLR8 (G/U-rich ssRNA) and TLR3 recognize dsRNA. Another group of PRRs is located in the cytoplasm and includes RIG-I, which has been shown to interact with short 5′-triphosphate containing dsRNA (5). In contrast, another member of this group, MDA5, recognizes long dsRNA, while dsRNAs more than 30 b.p. in length are substrates of PKR (37). It was also shown that NOD-like receptors (NLRP3, NOD2) can recognize viral ssRNA (9, 10). The main signal cascades initiated by PRR, and triggered by exogenous nucleic acids, pass through IRF3 or IRF7 proteins. So, the study of the involvement of IRF3/7 in isRNA signaling is important for the identification of pathways involved in isRNA signaling.

It was shown (28), that isRNA without any transfection reagent it does not have an antiproliferative effect on tumor cell lines and does not activate the secretion of cytokines after intravenous administration to mice. This allows us to conclude, that isRNA activate some cytosolic or endosomal sensors. Therefore, we used cationic liposomes as transfection reagent to deliver isRNA inside cell.

It is also should be noted, isRNA recognition in the cell could be similar to the recognition of the vital RNA in case of viral infection resulting in the formation of the antiviral state. During binding of virus to the cell, viral envelope proteins interact with cell membrane, but viral RNA does not. After fusion, viral double-stranded RNA is released into the cellular cytoplasm or is synthetized in the cytoplasm depending on the type of the virus. Therefore, viral dsRNA is recognized by cytoplasmic or endosomal receptors and not by cell surface receptors. Similarly, isRNA is also recognized by PRR inside cell and triggers immune reactions (39).

Based on literature data (5, 7–10, 36, 37) we selected RIG-I, MDA5, NOD2, and PKR, and interferon regulatory transcription factors IRF3/7 as potential mediators or sensors of isRNA antiproliferative activity. We show (Table 3), that KB-3-1 and A549 cells have a high level of *PKR* mRNA and medium levels of *IRF3, MDA5*, and *RIG-I* mRNAs. Thus, based on the mRNA levels, PKR, IRF3, MDA5, and RIG-I can participate in the recognition of isRNA or/and isRNA signal transduction in both cell lines. Using silencing of these genes by shRNA, we show that isRNA implements its antiproliferative activity through PKR and RIG-I sensors in KB-3-1 and A549 cells. We also show that RIG-I and PKR receptors play a major role in the induction of IL-6 synthesis in A549 cells in response to isRNA.

**Figure 4:**
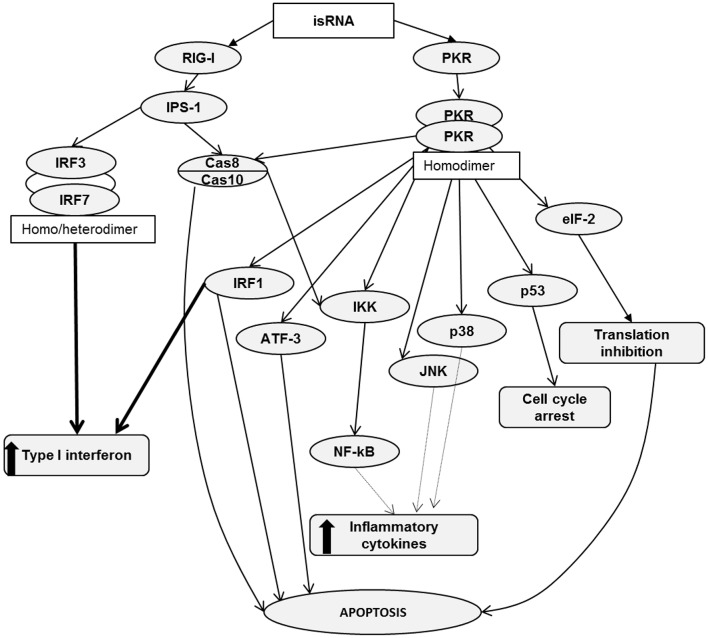
RIG-I and PKR signaling pathways. Activation of RIG-I/PKR signaling by RNA ligands in cancer cells induces different mechanisms, leading to apoptosis, cell cycle arrest, type I interferon and inflammatory cytokine production.

It is known that RIG-I recognizes RNA depending on sequence, length, single- or double-stranded structure and the presence of 5′ cap or 5′triphosphate (5′ppp). However, the variety of RNA sequences and structures is large enough that there is not enough comprehensive data allowing the assignment of certain molecules to agonists or antagonists by default. It has been reported that RIG-I signaling is not activated by 5′ capped RNA or RNA without 5′ppp or 5′ diphosphosphate (5′pp) (40–43). In addition, RIG-I signaling can be triggered by panhandle or bulge/loop RNA structures with blunted 5′ppp (44). RIG-I can also recognize synthetic dsRNA, for example poly (I:C) (45). Despite the fact that the studied isRNA did not have 5′ppp or 5′pp, it still activated RIG-I signaling. Apparently, the key elements that are important for activation of signaling are the motives of the sequence of isRNA, as previously we showed that certain sites of the sequence are important for the antiproliferative effect of isRNA and the substitutions introduced therein block the antiproliferative effect (29). It is also known that effective activation of RIG-I requires dsRNA from 10 to 300 b.p. in length, containing no mismatches near the blunt ends (5). In terms of length, isRNA fits well with the known RIG-I substrates, because isRNA has 22 b.p. Possible scenarios for the activation of cellular cascades under the action of isRNA, according to the obtained data and data known from the literature, are shown in Figure 4.

RIG-I consists of two N-terminal caspase activation and recruitment domains (CARDs), a central DExD/H box RNA helicase ATP-hydrolyzing domain and a C-terminal domain (CTD) (46). Based on literature data, it can be assumed that binding of isRNA to RIG-I leads to a conformational change in CARDs, which facilitates the association with an adaptor protein IPS-1 (IFNβ promoter stimulator 1, also known as MAVS). Then, through a number of mediators, the signal proceeds to caspase 8/10, which induce apoptosis and, through IKK family serine kinases, activate NF-κB signaling, which triggers the production of type I interferons and inflammatory cytokines. In particular, A549 cells secrete IL-6. Another possible pathway leading to type I interferon production passes through interferon regulatory transcription factors 3/7, IRF3/7. We did not find noticeable levels of *IRF7* expression in KB-3-1 and A549 cells. We also showed that *IRF3* silencing does not affect the antiproliferative effects of isRNA in KB-3-1 and A549 cells, but decreased the IL-6 inducing activity of isRNA in A549 cells. Perhaps the low level or lack of expression of this regulator in the studied cell lines is a reason why this line, unlike PBMC, does not secrete type I interferon in response to the action of isRNA. Thus, it seems likely that IRF3/IRF7 is not directly involved in the antiproliferative action but plays a role in isRNA signaling in these cells, which triggers the synthesis and secretion of interferons and cytokines.

PKR is a serine-threonine kinase that consists of N-terminal dsRNA binding domains (dsRBD) and a C-terminal kinase domain containing major phosphorylation sites (47, 48). PKR recognizes long dsRNA in a non-sequence specific manner, and activation of PKR by dsRNA does not require a 5′-ppp (49). A number of authors have shown that PKR recognizes dsRNA not shorter than 30 b.p. (50–53), but activation of PKR by dsRNA containing 19–21 b.p. has also been reported (7, 54). We showed that isRNA does activate PKR, because PKR silencing entirely abolished both the antiproliferative and IL-6 inducing activities of this molecule. It should be noted that blunt end dsRNAs activate PKR less potently than those with protruding ends (7) and, importantly, isRNA has 3-nucleotide 3′-overhangs. Previously, we have shown that shortening of the protruding ends of isRNA or nucleotide replacement of A/U by G/C blocks the antiproliferative effect of isRNA (29).

We can assume that PKR binding with isRNA leads to its homodimerization and rapid autophosphorylation by analogy with similar molecules (55, 56). Then, activated PKR phosphorylates translation initiation factor eIF2α, and phosphorylated eIF2α prevents the formation of the translation initiation complex eIF2-GTP-Met-tRNAi. Such inhibition of protein synthesis limits cell proliferation and induces apoptosis in cancer cells (12, 57, 58). PKR can also act through several other signal transduction pathways, including Cas8/10 (59), IRF1 (60), and p53 (61). PKR activation either mediates apoptosis or interferon production or both, and thereby reduces the proliferation rate. PKR is able to activate the transcription factor ATF-3, which is involved in apoptosis (62). Activation of NF-κB, JNK, and p38 by PKR leads to the production of inflammatory cytokines (63–67). Thus, there are many PKR-triggered pathways that can lead to IL-6 production and inhibition of cancer cell proliferation. It was shown (68), that the function of PKR, independently of translation inhibition, is critical for type I interferon production downstream of MDA5 and upstream of MAVS. It has also been shown (68) that after viral infection, PKR co-immunoprecipitates with RIG-I and MDA5 independently on RNA binding. It also should be noted that RIG-I and PKR sensors are upstream proteins in these signal transduction pathways. Previously, we showed that penetration into the cell is necessary for the antiproliferative effect of the isRNA. This indicates that the sensors that trigger the signaling pathways leading to a decrease in the rate of cell growth are localized in the cytoplasm rather than on the cell membrane (29).

PKR- and RIG-I-triggered pathways intersect at two points: the first one is Cas8/10, the activation of which leads to apoptosis and IKK activation, and the second IKK itself, because IKK can be activated by RIG-I through Cas8/10 only, but PKR activates IKK both in a Cas8/10-dependent or Cas8/10-independent manner. IKK activation by RIG-I or PKR leads to the synthesis of proinflammatory cytokines through the mediator NF-kB. This comparison of RIG-I and PKR functions is in a good agreement with the data obtained in this study and data previously obtained on the *in vitro* and *in vivo* effects of isRNA (28–30, 69).

Previously, we have demonstrated that the antiproliferative effect of isRNA on KB-3-1 cells is associated with a decrease in the growth rate, and not with the induction of apoptosis (29). It was found that isRNA only slightly increases the number of apoptotic and dead cells in comparison with controls, but increases the number of cells in G0/G1 phase and reduces the number of cells in G2/M phase (29). Considering the results obtained in this study, we can conclude that signal transduction pathways triggered by isRNA/RIG-I or isRNA/PKR interactions lead mostly to type I interferon synthesis, translation inhibition and cell cycle arrest, but not to the induction of apoptosis, or synthesis of proinflammatory cytokines.

## Data Availability Statement

All datasets generated for this study are included in the article/Supplementary Material.

## Author Contributions

EC conceived and designed the experiments. MIZ performed the experiments. MIZ and EC analyzed the data. VV and EC contributed reagents, materials, and analysis tools. MAZ and EC wrote the paper.

### Conflict of Interest

The authors declare that the research was conducted in the absence of any commercial or financial relationships that could be construed as a potential conflict of interest.
