# Supplementary material for: Molecular Mechanism of the Antiproliferative Activity of Short Immunostimulating dsRNA
Source: Front Oncol. 2019 Dec 20;9:1454. doi: 10.3389/fonc.2019.01454 (PMC6933605; doi:10.3389/fonc.2019.01454)
Supplement: Supplementary file 1 [file Table_1.DOCX]

Supplementary Material
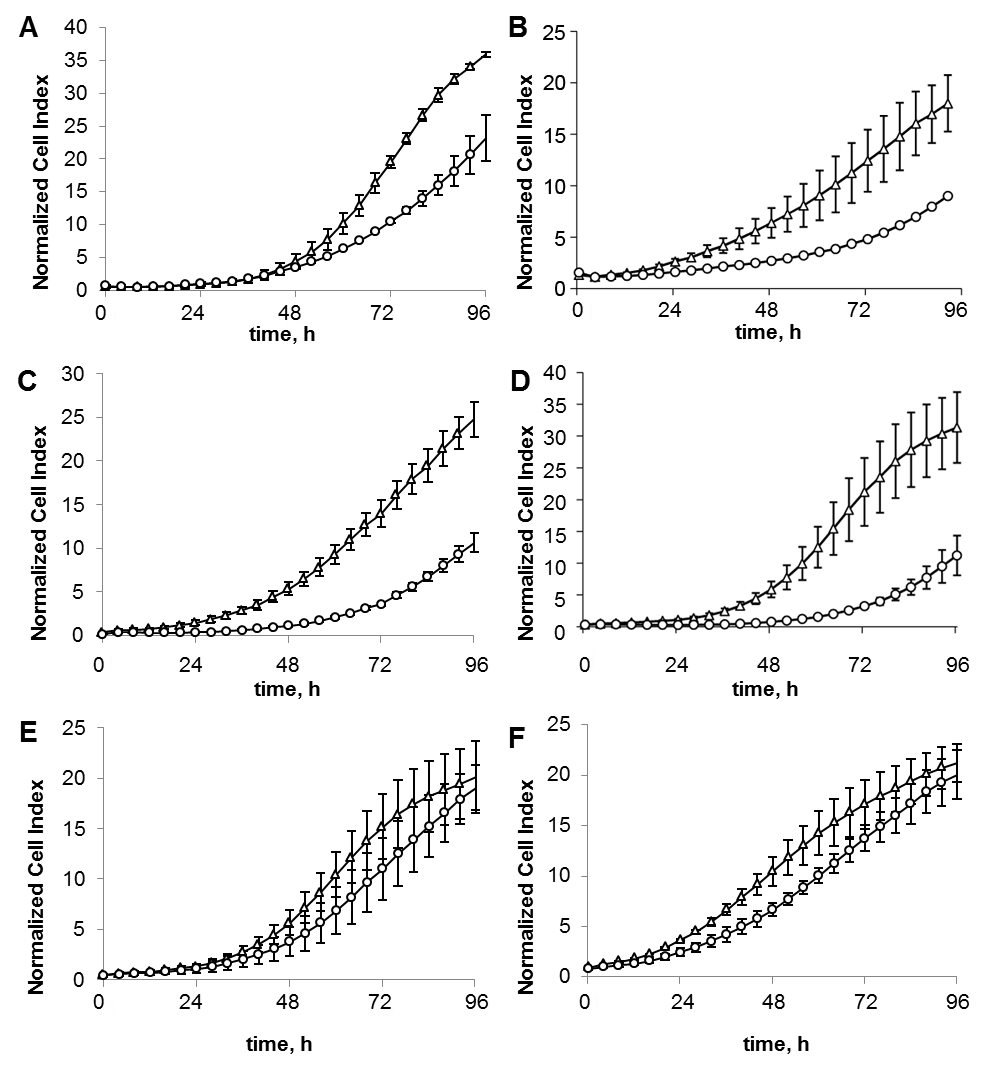


**Supplementary Figure S1.** The effect of isRNA/2X3-DOPE complexes (ο) or 2X3-DOPE alone (∆) on the proliferation of parent A549 cells (A), and sublines A549-Scr (B), A549-MDA5 (C), A549-IRF3 (D), A549-RIG-I (E), A549-PKR (F). After transfection, the relative number of living cells was measured every 4 h for 96 h. The number of living cells 4 h after transfection was set at 1. Experiments were performed in four repeats. The data represent means +/- SD.
